# Lipidomics Analysis Reveals the Effects of Docosahexaenoic Acid from Different Sources on Prefrontal-Cortex Synaptic Plasticity

**DOI:** 10.3390/nu17030457

**Published:** 2025-01-27

**Authors:** Zude He, Wei Xiong, Yue Yang, Yifan Zhang, Boying Li, Fuqing Wang, Yixuan Li, Ran Wang, Yanan Sun

**Affiliations:** 1 Key Laboratory of Functional Dairy, Co-Constructed by Ministry of Education and Beijing Municipality, College of Food Science & Nutritional Engineering, China Agricultural University, Beijing 100083, China; hezudee@126.com (Z.H.); d202380962@hust.edu.cn (Y.Z.); 2 Key Laboratory of Precision Nutrition and Food Quality, Department of Nutrition and Health, China Agricultural University, Beijing 100193, China; xiongwei910702@126.com (W.X.); 100403yang@sina.cn (Y.Y.); lboying@126.com (B.L.); liyixuan9735@126.com (Y.L.); 3 Food Laboratory of Zhongyuan, Luohe 462300, China; 4 Tibet Tianhong Science and Technology Co., Ltd., Lhasa 850000, China; fq7963@163.com

**Keywords:** DHA intervention, synaptic plasticity, PFC, lipidomics, lipid composition

## Abstract

Background: Docosahexaenoic Acid (DHA) is an extensively used nutrition supplement in dairy food because of its beneficial effects on cognition. To find an effective DHA intervention for the synapses in the cortex during this period, this study aimed to use targeted lipidomics to evaluate the lipid composition of prefrontal-cortex (PFC) tissue in different DHA interference methods. Methods: Analyzed samples were taken from interfering feeding Bama pigs (BPs) (3 months) fed with soybean oil (Group B), blended oil (Group M), naturally DHA-supplemented milk with blended oil (Group OM), and DHA from fish oil (FO) with blended oil (Group Y). We also examined the protein expression levels of BDNF, GAP43, and MBP. Results: The lipidomics analysis identified 80 different related negative-ion lipid content and filtered the biomarker lipids in PFC tissue. We observed significant lipid composition changes between group Y and other groups, especially for content levels of phosphatidylcholine (PC), phosphatidylethanolamine (PE), phosphatidylinositol (PI), phosphatidylserine (PS), and sphingomyelin (SM). The same observations were made from mRNA and protein expressions related to lipid transportation, phosphatidylserine (PS) synthetase, and synaptic plasticity in PFC tissues between group Y and other groups, including the mRNA expression levels of CD36, BDNF, and PTDSS1. The analysis of protein expression levels showed that the metabolism mode of DHA intervention from FO benefited the PFC, PS metabolism, and PFC synaptic plasticity of infants. Conclusions: The results highlight further prospects for the DHA intervention mode, which provides new routes for other studies on polyunsaturated-fatty-acid (PUFA) interference for infants.

## 1. Introduction

Docosahexaenoic acid (DHA) is a polyunsaturated fatty acid (PUFA) that can be obtained from fish oil (FO), milk, and algal oil [1]. DHA has several functions that can benefit children and seniors. Moreover, it is among the most efficient nutritional supplements for the brain in early life [2] and plays a role in the synthesis of membrane lipids and proteins [3]. In previous studies, intervention of DHA was found to improve the development of the myelin sheath and synaptic connectivity, which can effectively inhibit brain degeneration [4,5,6,7]. Furthermore, few studies have focused on lipid metabolism with DHA intervention from various sources, much less on those about the relationship between DHA from various origins and synaptic development in the brain [8]. Furthermore, DHA could also promote cholesterol metabolism, which may reduce the level of inflammatory factors in blood [9,10]. After metabolism, orally supplied DHA is transported from the circulating blood and passes through the blood–brain barrier (BBB) by the major facilitator superfamily domain-containing protein 2a (MFSD2A) and CD36 (thrombospondin receptor) [11,12]. Especially for seniors, DHA supplementation for the brain has been found to effectively alleviate nerve damage [13]. In recent years, PUFAs (especially for DHA & EPA) have been widely used for nutritional fortification of dairy products, which have great potential as a functional food group to improve children’s cognitive activity and memory [14]. However, the DHA synthesis rate of the human body is relatively low, although it can be synthesized from alpha-linolenic acid in the body [15]. Thus, the organism still necessarily relies on food intake to supplement DHA.

Lipids are indispensable nutrition for the cortex and are the main synthetic precursor of phospholipids (PLs) and sphingomyelin (SM). Based on the previous study, we know that the PUFAs in the brain can be used for therapies for neurodegenerative diseases [16]. This is especially true for PUFAs like DHA and EPA, which are the main lipid-related nutritional supplements in dairy and sea products [17,18,19]. ω-3 fatty acid is crucial for optimal brain functionality because DHA is the principal omega-3 FA in cortical gray matter that may influence degenerative diseases [20]. Thus, suitable supplementation of DHA could reduce the risk of neurological diseases [21]. Compared with ω-3 fatty acid, the function of ω-6 fatty acid also cannot be neglected. The ratio of ω-6 and ω-3 fatty acids is crucial for brain and muscle development [22]. Depression and other symptoms of abnormal functions of brain monoamine systems were found in rat and mouse models with low levels of DHA in the brain [22]. The cerebral cortex is the outer layer of the brain’s hemispheres, responsible for integrating sensory input and processing information [23]. It plays a crucial role in cognitive functions. During childhood, the cerebral cortex undergoes differential development, supporting the acquisition of various functions [24]. It also regulates sleep and sensory information processing, further emphasizing its significance [25,26]. Nevertheless, cerebral cortex thinning could cause degenerative disease that is characterized by a decrease in cognitive dysfunction and poor memory [27]. Despite the hippocampus’s indispensable role in memory encoding and determining its formation, the cortex is responsible for its stability [28]. For the whole brain, DHA is first transported into the cortex [29], so we must assess the changes of related components in the cortex tissue. Take the prefrontal cortex (PFC) as an example. Regardless of DHA’s functional effects on cognitive abilities [30], a study found that high intake of DHA could cause the incomplete structure of the retina [31]. Synaptic plasticity is also important for memory consolidation in the cortex [28], and it plays a critical role in long-term memory [32]. Furthermore, a study found that DHA affected the synaptic plasticity and signal transduction in the brain [33]. Therefore, we focused our study on the effects of synaptic plasticity in the PFC with DHA supplementation. Based on the research need for suitable DHA supplements, seeking proper intervention strategies for DHA to promote synaptic plasticity in the cortex has become an urgent task. This study concentrated on the cortex tissue of the Bama pig (BP) accordingly, which has highly similar features to those of humans. After establishing related animal models to make DHA interventions from various origins and combinations, we analyzed the lipid composition changes in different DHA supplementation methods, which also offer new ideas for DHA nutrition interference.

According to the previous study, taking DHA and uridine could enhance the formation of synapses [34]. Also, a study found that phosphatidylserine (PS) participated in the modification of synaptic ultrastructure [35]. The PFC is highly enriched with DHA, and DHA supplements can expand the PS pool, which influences PS-dependent signaling and protein function [36]. Thus, we hypothesized that DHA interference can effectively promote the synthesis of PS, which may improve the synaptic plasticity and thus promote cognition and memory.

Nonetheless, DHA supplements from various sources may have different influences on the cortex. As a PUFA that can be gained from deep-sea FO and dairy, it also composes the PL bilayer, which can promote the fusion and receptor activities of the cell membrane [37,38]. Nevertheless, most studies have concentrated on the neurodevelopment and neuroprotection mechanism of DHA, instead of analysis of interference modes with DHA from various origins [39]. A study also concentrated on the bioavailability of different DHA formations [40], but they neglected the source factor. Based on these studies, this study accurately analyzed the metabolism content of related lipids in cortex tissue from BPs, which have more similar brain structures to human beings, through the liquid chromatography and mass spectrometry technique (LC-MS).

This study aimed to evaluate the effect of lipid content changes with the interference of DHA from various sources on lipid metabolism, filtering the biomarkers of PLs. These results show that various sources of DHA induced PFC lipid content changes, improved the synaptic plasticity, and revealed the relationships between PS synthesis and synaptic plasticity in the PFC.

## 2. Materials and Methods

### 2.1. Materials

Naturally DHA-supplemented cow’s milk was provided by Inner Mongolia Yili Industrial Group, Co. Ltd., and was used as feed for group OM. The composition of this milk is shown in Table 1. All the milk for mixing feed was Jindian pure milk (Yili, Hohhot, China), which lacks DHA, except for group Y. The daily intake volume of DHA was controlled at the level of 11 mg/kg/d for each pig. The blended oil was mixed with 50% soybean oil (Huifu, Sanhe, China), 25% rapeseed oil (Shanrun, Changsha, China), 9% peanut oil (Shanrun, Changsha, China), 9% palm oil (Tianyijia, Shanghai, China), 5% corn oil (Jinlongyu, Shanghai, China), and 2% cottonseed oil (Tianyijia, Shanghai, China), whose proportion of ω-3:ω-6 was 6.6:1, which was approximately equal to that in soybean. The volume of FO (Yili, Hohhot, China) in group Y was calculated based on the animals’ weight. We calculated the feed without DHA by maintaining the calorie level at 3270 kcal/kg/d. The formulation of the feed is shown in Table 2.

### 2.2. Animals

Twelve 4-week-old male BPs were purchased from Jiaxiang-Shihui Laboratory Animal Breeding Center (Shandong Province, China), and their weight range was 3.25 ± 0.25 kg. Animals were housed in a controlled environment (40% humidity, temperature 23 ± 3 °C, 12 h cycle light/dark, and one pig per cage) at the Animal Science Research Center in Beijing. The pigs were randomly divided into one of four groups and received interference with pig feed without DHA as follows: (i) Blank Group (Group B, mixed feed without DHA with soybean oil and Jindian pure milk); (ii) Model Group (Group M, mixed feed without DHA with soybean oil blended oil and Jindian pure milk); (iii) Original Milk DHA Group (Group OM, mixed feed without DHA with blended oil and naturally DHA-supplemented milk, 11 mg/kg/d of DHA); (iiii) FO DHA Group (Group Y, mixed feed without DHA with blended oil, Jindian pure milk and FO, 11 mg/kg/d of DHA). All volumes of feed and milk were calculated based on weights. The feed interference was operated for 3 months.

The study was conducted according to the national standards outlined in “Laboratory Animal Requirement of Environment and Housing Facilities” (GB149250–2010) [41]. The protocol for animal experiments was approved (approval no. AW42403202-5-1) by the Ethical Committee of Experimental Animal Care of China Agricultural University.

### 2.3. Sample Collection

After 13 weeks of feeding, pigs were anesthetized with 2% pentasorbital sodium (*w*/*v*) to minimize their pain during operation. We then cut the head and sectioned the skin tissue. We collected the whole brain tissue after cutting off the skull of the pig, and then we weighed the whole brain. After these operations, we separated the PFC tissue and removed other parts. With the addition of liquid nitrogen into a mortar, the tissues were divided into 1-mm granular material evenly, which was also saved in a −80 °C fridge.

### 2.4. Lipid Extraction

We accurately weighed 500 mg of PFC tissue and added 1 mL of extraction liquid (600 μL N-hexane (HPLC, Sinopharm, Beijing, China) and 400 μL of isopropanol (HPLC, Sinopharm, Beijing, China)) in the saving tube, with the usage of a Biological Sample Homogenizer (Bertin, Paris, France) on 7500 r for 30 s three times. Then, we put the homogenate into a 1.5 mL RNase-free centrifuge tube, with 2500× *g* for 5 min in the high-speed centrifuge (Eppendorf, Hamburg, Germany), and we suctioned the supernatant with the mixture of anhydrous saturated sodium sulfate solution. After approximately 1 h of sedimentation extraction, we extracted the upper liquid and dried it with CO_2_ gas by a nitrogen-blowing instrument (Beijing Yousheng United Technology Co., Ltd., Beijing, China). The white materials at the bottom were the extracted lipids of the cortical tissue.

### 2.5. Targeted Lipidomics Analysis Using LC-MS

Analysis of lipids was performed with the UPLC-Q-TOF (Agilent, Santa Clara, CA, USA). The column temperature was set to 55 °C, the flow rate was controlled at 0.3 mL/min, and the injection volume was 2 μL. The specific elution procedure is shown in Table 3, and the mass spectrometry setting conditions are shown in Table 4.

### 2.6. Calculation and Normalization of Peak Area in Lipidomics

The data were collected using Agilent MassHunter Workstation Data Acquisition Version B.10.001 software, and then features were identified and statistically analyzed using MassHunter and Mass Programmer Professional software, respectively.

### 2.7. Bioinformatics Analysis

The heatmap was mapped by R Studio with Pheatmap Package, and the chordal map and the Venn map were made using Origin 9.1 (OriginLab, Northampton, MA, USA). MetaboAnalyst 6.0 online software was used for data normalization. Network analysis of the targeted lipids was based on the debiased sparse partial correlation (DSPC) network. The pathway analysis was analyzed according to the Sus Scrofa (pig) (KEGG) database. Moreover, the enrichment analysis of the targeted lipids was analyzed with the lipids feature and based on the RaMP-DB and Sub_Class pathway.

### 2.8. RNA Extraction and cDNA Conversion

Total RNA was extracted from the PFC tissue, adding 1 mL of Trizol (Thermo Scientific, Waltham, MA, USA) for each sample. After homogenization by a Biological Sample Homogenizer (Bertin, Paris, France), the lysate was resuspended with 75% ethanol (Zhiyuan, Tianjin, China) and isopropanol (Zhiyuan, Tianjin, China) and centrifugated for 10,000× *g* for 10 min in a high-speed centrifuge (Eppendorf, Hamburg, Germany). Second, detecting the RNA concentration by Nanodrop One (Thermo Scientific, Waltham, MA, USA), we dissolved 1000 ng of RNA and converted it to complementary DNA (cDNA) using the 5 All-In-One RT Master Mix (ABM, Richmond, BC, Canada), according to the manufacturer’s protocol.

### 2.9. Gene Expression Analysis Using Real-Time PCR

This essay focused on the gene expression and was analyzed through RT-qPCR using the SYBR qPCR Mix (YEASEN, Shanghai, China), according to the manufacturer’s protocol. Primer sequences are listed in Table 5. The QuantStudio™ 5 Real-Time PCR Systems (Applied Biosystems, Carlsbad, CA, USA) was used for all essays. We used the housekeeping gene GAPDH as the normalized gene to calculate the mRNA expression levels of other genes.

### 2.10. Protein Extraction and Analysis

Total samples were homogenized with RIPA solution (Beyotime Institute of Biotechnology, Shanghai, China), which also included protease and phosphatase inhibitors (Beyotime Institute of Biotechnology, Shanghai, China), whose lysate was broken down by a cell crusher at 35% for 30 s. The homogenate was transferred to a 1.5-mL centrifuge tube, with the collection of upper liquid being done after a 12,000× *g* 15 min centrifugation in a desktop low-temperature high-speed centrifuge (Eppendorf, Hamburg, Germany). The protein was separated successfully. The protein abundance was adjusted using a BCA kit (Solarbio, Beijing, China) with a microplate reader (Agilent BioTek, Winooski, VT, USA). The mixture of samples was then boiled with 5X Protein Loading Buffer (Solarbio, Beijing, China) for 5 min in a Dry Bath Incubator (LEPARD, Beijing, China), and then we stored the samples in a –20 °C fridge.

### 2.11. Western Blotting

The samples were subjected to SDS-PAGE (CWBIO, Taizhou, China), and the proteins were transferred to the PVDF membrane (Merk Millipore, Tullagreen, Ireland) after electrophoresis (Bio-Rad, Hercules, CA, USA). The membranes were blocked with 5% skimmed milk in PBST (Solarbio, Beijing, China) at 25 °C for 2 h. After incubating with primary antibodies at 4 °C for 12 h, the primary antibodies were as follows: GAPDH Monoclonal Antibody (Proteintech, Wuhan, China, 1:10,000), BDNF Monoclonal Antibody (Proteintech, Wuhan, China, 1:500), GAP43 Polyclonal Antibody (Proteintech, China, 1:2000), and Myelin Basic Protein Polyclonal Antibody (Proteintech, Wuhan, China, 1:1000). The membranes were incubated with HRP-labeled goat anti-rabbit/mouse IgG (H+L) antibodies (Beyotime Institute of Biotechnology, Shanghai, China, 1:5000) at 25 °C for 1 h after a 30-min washing with PBST. Before imaging by the X-ray system (Amersham Imager 600, General Electric Company, Boston, MA, USA), we washed the membranes with PBST for half an hour.

### 2.12. PTDSS1 ELISA Kit

The samples were added in saving tubes with 1 mL of PBS buffer (Solarbio, Beijing, China) and were broken by a cell crusher at 40% for 30 s. Then, we detected the enzyme volumes of PTDSS1 according to the protocol of Pig PTDSS1 Elisa Kit (Fushengshiye, Shanghai, China). We used the BCA kit (Solarbio, Beijing, China) to calculate the sample volumes of PTDSS1.

### 2.13. Statistical Analysis

Significant differences between the groups were established by ANOVA and Waller–Duncan comparisons (SPSS Software version 21.0, Chicago, IL, USA). Another statistical analysis was conducted by GraphPad Prism 8 (GraphPad, San Diego, CA, USA). The level of statistical significance was set at *p* < 0.05 between different groups, revealing a significant difference.

## 3. Results

### 3.1. DHA Intervention from FO Significantly Changes the Content of Related PLs and Ceramide inPFC Tissue

**Figure 1:**
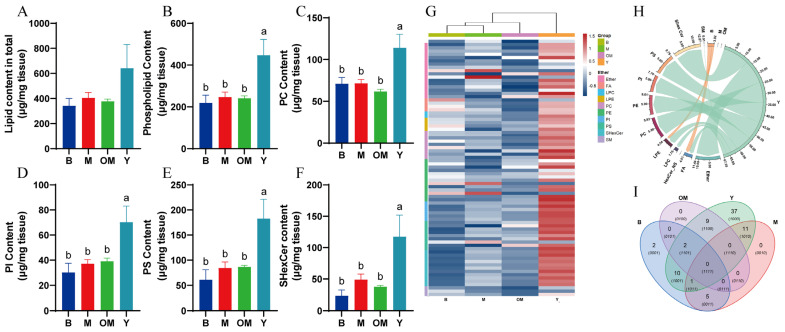
Comparison of total lipid contents in PFC tissues from different groups: (**A**) Lipid contents in total. (**B**) PL content. (**C**) Total PC content. (**D**) Total PI content. (**E**) Total PS contents. (**F**) Total SHexCer contents. (**G**) Heatmap. (**H**) Chord plot. (**I**) Venn diagram Analysis. Data are shown as the mean ± SEM, *n* = 4. The significance of the differences was estimated by one-way ANOVA. a, b: Represent significant differences (*p* < 0.05). PC: phosphatidylcholine; PI: phosphatidylinositol; PS: phosphatidylserine; SHexCer: sulfated hexosyl ceramide.

To evaluate the effects of weight to lipid content levels, we statically analyzed the body and brain weight (Table 6), and no significant differences were found. PLs and ceramide are important nutrition lipids for the PFC that closely correlate with the amelioration of the damage to cognitive functions. First, we investigated the total content levels of targeted lipids in PFC tissues classified by detailed types. The differences in the lipid content of PFC tissue supplied with different DHA interference methods were compared, as shown in Figure 1A. The highest total lipid content was in the cortex of group Y (641.60 μg/mg), followed by group M (404.22 μg/mg), group OM (377.74 μg/mg), and group B, which had the lowest total lipid content (341.69 μg/mg), but no significant differences were found between each group (*p* > 0.05). In Figure 1B, we also found that the highest PL content level was in group Y (387.86 μg/mg), followed by group M (247.94 μg/mg), group OM (242.68 μg/mg), and group B (220.821 μg/mg). Significant differences were found between group Y and other groups (*p* < 0.05).

We compared five related lipid categories in the PFC tissue, and the results are presented in Figure 1C–F. With the intervention of DHA from FO (Group Y), the content levels of phosphatidylcholine (PC), phosphatidylethanolamine (PE), phosphatidylinositol (PI), PS, and sulfated hexosylceramide (SHexCer) showed significant differences with other groups (*p* < 0.05). The lipid levels of group M and group OM were slightly higher than those of group B, except for the PC content level, but there were no significant differences between group M or OM and group B (*p* > 0.05). In the following content, the detailed names of lipid components are given as abbreviations. The first two numbers denote the carbon chain length and unsaturated bond location, and the character denotes the abbreviated lipid name. This research analyzed the negative mode data of the results. The evaluation includes four groups, each with three samples.

### 3.2. Lipidomics Analysis Revealed That DHA Supplements Upregulated the Levels of PLs and Sphingomyelin in the PFC Tissue of Pigs

To further discover the metabolic profile of the cortex and DHA-supplemented methods, we analyzed the lipidomics data using LC-MS. To compare related PLs in different interference methods, the results determined by LC-MS reveal 80 different negative ion lipids, which could be classified into 10 groups. As shown in Figure 1G, we identified significant inter-group differences in lipid levels and the abundance of 10 clusters: ether-related lipids (Ether), lysophosphatidylcholine (LPC), Lysophosphatidylethanolamine (LPE), PC, PE, PI, PS, ceramides (Cer), sphingomyelins (SM), and SHexCer. Treatments of group Y significantly increased the levels of PC, PE, PI, PS, and SHexCer. Correlation analysis of lipids in the pig PFC showed that 68 metabolites in group Y were significantly upregulated in comparison with other groups, and that seven main clusters formed the separate subcluster. Among them, FO DHA might influence the synthesis process of PLs and sphingomyelin, which might promote the cell membrane functions that aid in cognitive functions.

To analyze the degree and proportion of the relationship between each group and its components, especially for the lipid co-regulatory relationship and the correlation between lipid subclasses, we created a chord plot, shown in Figure 1H, which indicates the lipid molecule pairs. The correlation between lipid subclasses in group Y presented differences in almost all categories of related lipids. In the chord plot, 10 metabolite classes were integrated around the circle. Moreover, the connections between metabolites and groups revealed the degree of connections. Connections with green and orange colors presented positive and negative correlations, respectively. The arc size in different groups indicated the abundance of lipid metabolites. Take group Y as an example; its arc was wide, meaning that it had more special lipid metabolites, which revealed that FO DHA intervention changed its lipid composition. In group Y, 37 candidate metabolites had special features, while group B only had two special features.

These results show that DHA interference in group Y could effectively increase the abundance and content levels of PLs and sphingomyelin compared with groups B and M. Therefore, we hypothesized that the DHA supplements may change the lipid composition in the PFC with the increasing level of related PLs like PS, which can benefit membrane functions in glial cells, as well as synaptic plasticity.

### 3.3. Changes in PC Content in PFC Tissue

**Figure 2:**
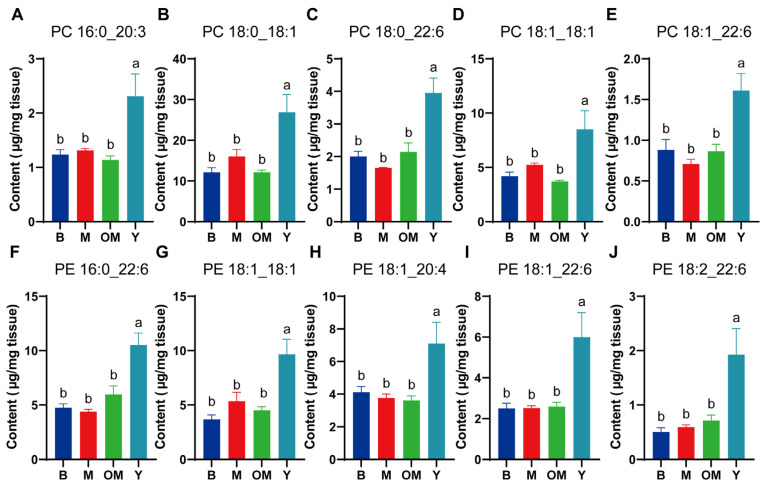
The content levels of PC (**A**–**E**) and PE (**F**–**J**) in different groups. Data are shown as mean ± SEM, *n* = 3. The significance of the differences was estimated by one-way ANOVA. a, b: Represent significant differences (*p* < 0.05). PC: phosphatidylcholine; PE: phosphatidylethanolamine.

Different interference methods affected the content levels of PLs in PFC tissue. This part analyzed the related data of PC (Figure 2A–E), which revealed that those PC indicators in group Y had significant differences with other groups. After Waller-Duncan analysis, significant differences were found in PC 16:0_20:3, PC18:0_18:1, PC18:0_22:6, PC 18:1_18:1, and PC18:1_22:6 between group Y and group M, which showed that DHA from FO could sharply increase PC levels. Moreover, we observed significant differences in the content levels of PC16:0_20:3, PC18:0_18:1, PC18:0_22:6, PC18:1_18:1, and PC18:1_22:6 between group Y and group OM, which presented effective transaction efficiency from FO DHA to PC.

### 3.4. Changes in PE Content in PFC Tissue

Among the most vital components in cell membranes, PE also determines the condition of signal conduction in the synapse of PFC tissue. Based on its functions, this part included a Waller–Duncan analysis for five related materials (Figure 2F–J), which presented significant differences between group Y and group M for content levels of PE 16:0_22:6, PE 18:1_18:1, PE 18:1_20:4, PE 18:1_22:6, and PE 18:2_22:6, which showed the PC structural component changes with DHA supplementation. Furthermore, similar significant differences between group Y and group OM were also found, revealing that DHA from FO could effectively promote the synthesis of PC.

### 3.5. Changes in PI Content in PFC Tissue

**Figure 3:**
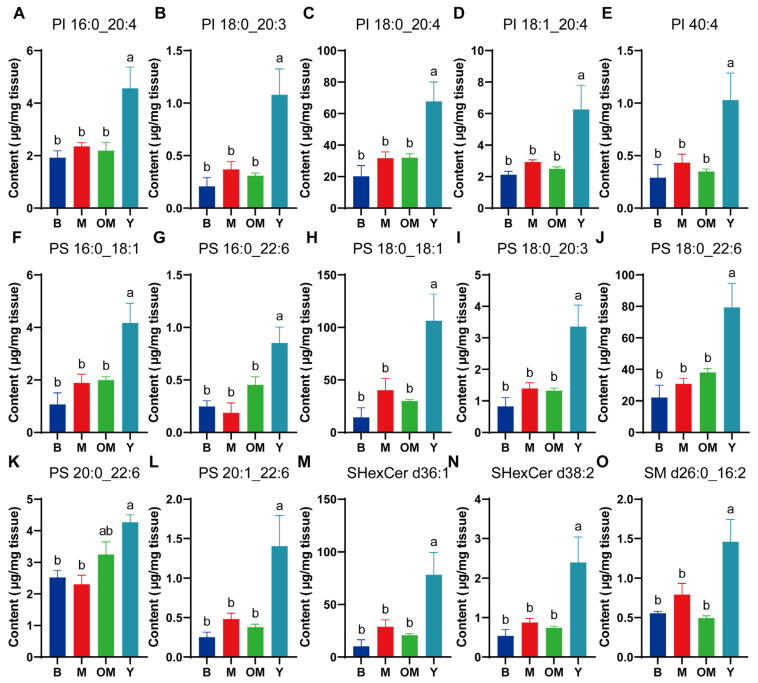
The content levels of PI (**A**–**E**), PS (**F**–**L**), SHexCer (**M**,**N**), and SM (**O**) in different groups. Data are shown as mean ± SEM, *n* = 3. The significance of the differences was estimated by one-way ANOVA. a, b: Represent significant differences (*p* < 0.05). PI: Phosphatidylinositol; PS: Phosphatidylserine; SHexCer: Sulfated hexosyl ceramide; SM: Sphingolipids.

Differently from PE, PI is the main component of the organelle membrane in neurons, regulating the development of the membrane pore. In this part, we also chose six PIs for a one-factor Waller–Duncan analysis, and significant differences were found in PI 16:0_20:4, PI 18:0_20:3, PI 18:0_20:4, PI 18:1_20:4, and PI 40:4 between group Y and group M (Figure 3A–E), which revealed that DHA from FO might promote the transportation function of membranes in PFC tissue. We also found similar significant differences between group Y and group OM, revealing that DHA supplements with FO might increase the synthesis degree of PI and promote the membrane functions of organelle.

### 3.6. Changes in PS Content in PFC Tissue

PS is a main component of membrane PLs, and it is also one type of derivative of soybean PLs that can be easily obtained through diet. It can improve cognitive and memory function by repairing cell damage and promoting signal conduction for neurotransmitters. This part analyzed nine types of related PS content levels that presented visible changes in PS-related levels between group Y and other groups. Based on this difference, this part also involved a Waller–Duncan analysis. Those lipid levels of PS 16:0_18:1, PS 16:0_22:6, PS 18:0_18:1, PS 18:0_20:3, PS 18:0_22:6, PS 20:0_22:6, and PS 20:1_22:6 showed significant differences between group Y and group M (Figure 3F–L), which revealed DHA supplements could promote the synthesis of PS. Moreover, we found significant differences between group Y and group OM for those levels of PS 16:0_18:1, PS 16:0_22:6, PS 18:0_18:1, PS 18:0_20:3, PS 18:0_22:6, and PS 20:1_22:6. This outcome also proved that DHA interference from FO could effectively raise the content level of PS lipids compared with those from milk.

### 3.7. Changes in SHexCer Content in PFC Tissue

As shown in Figure 3M,N, significant differences were found between group Y and group M in content levels of ShexCer d36:1 and SHexCer d38:2. Also, similar significant differences were found between group Y and group OM. These outcomes suggest that the upregulation of SHexCer by fish oil-derived DHA may enhance neuronal conductivity in the cortex.

### 3.8. Changes in SM Content in PFC Tissue

SM is a dominant component in myelin sheath synthesis in the brain, and it is also indispensable for biological regulation in brain cells and the process of cell differentiation and apoptosis. More importantly, SM is a critical component that consists of myelin sheath, and its deficiency might have negative effects on signal conduction. A significant difference was found for the content level of SM d26:0_16:2 between group Y and OM (Figure 3O), which revealed that DHA from FO might increase the utilization rate of the transfer process from DHA to SM in PFC tissue, which benefits the regulation activities in the cortex.

### 3.9. Network Analysis of Differentially Expressed Lipids in PFC Tissue

The results in Table 7 show that 10 different lipid metabolites in the PFC tissue were directly related. In the comparison of group B and Y, we filtered PI 18:0_20:3 as lipids that had nine interacting lipid metabolites, followed by PS 18:0_22:6 and SM d36:1 with seven interacting lipid metabolites. We found 47 metabolites that were directly related. Moreover, in group Y, PS 18:0_20:3 and PE 18:0_22:4 had seven interacting metabolites compared with group M. In group OM, SHexCer d36:1 had nine and SHexCer d 38:2 had eight edges compared with group Y. In this analysis, 41 lipid metabolites were directly related. The results illustrated that PS, PE, and PI were the first three strongest interacting metabolite classifications.

### 3.10. Pathway Analysis

**Figure 4:**
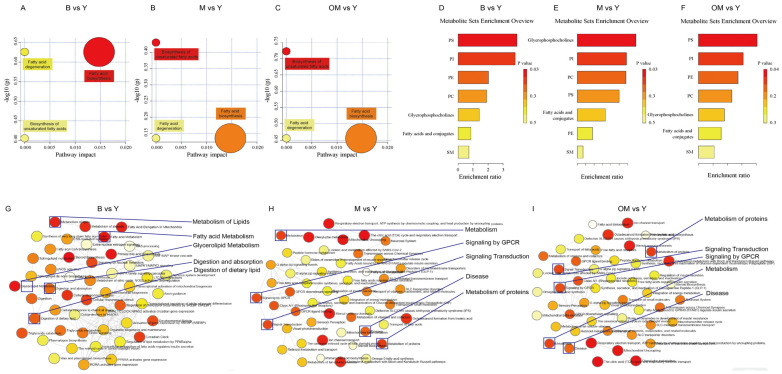
Pathway analysis based on differential species: (**A**) B vs. Y. (**B**) M vs. Y. (**C**) OM vs. Y. The node colors (yellow and red) are based on p-values (*Y*-axis, where yellow indicates higher p values; red indicates lower p values), and the node size determines impact values of the pathway (*X*-axis, where the larger the size, the higher the impact score). Enrichment Analysis of the related node based on Sub_Class database: (**D**) B vs. Y. (**E**) M vs. Y. (**F**) OM vs. Y. Partial Correlation Network integrating related metabolic functions, displaying interactions and betweenness centrality. (**G**) B vs. Y. (**H**) M vs. Y. (**I**) OM vs. Y. PC: phosphatidylcholine; PI: phosphatidylinositol; PS: phosphatidylserine; SHexCer: sulfated hexosyl ceramide.

Based on the following analysis, we performed a pathway analysis to gain a better understanding of the roles of the identified lipid metabolites in the synthesis and degeneration of fatty acid and unsaturated fatty acid, especially for PLs. As shown in Figure 4A–C, compared with group B and Y, we found that the interference mode in group Y could effectively increase the significancy of fatty acid biosynthesis with most metabolites. The samples in group Y and M actually showed that the interference in group Y effectively promoted the fatty acid synthesis with the most metabolites, but a significant difference was found in biosynthesis of unsaturated fatty acids. The same trend was also observed in the comparison of Group OM and Y.

### 3.11. Enrichment Analysis

To determine how lipid metabolism regulates other biological activities in PFC tissue, we evaluated the functions by calculating a partial correlation network. As shown in Figure 4G–I, in the comparison of group B and M, these data reveal that the metabolism of lipids, fatty acid metabolism, and digestion of dietary lipids had higher degrees of connection centrality scores than other components. Other comparisons with Group Y also presented the same trend on metabolism, metabolism of lipids, disease, metabolism of protein, and signal transduction, so we hypothesized that PLs have a higher enrichment ratio with the combination evidence of PS lipid content levels and enrichment analysis. To continue to discover the relevant relations, we calculated the enrichment ratio of different lipids based on the Sub_class database (Figure 4D–F). In the comparison between group B and Y, group OM and Y, we found that PS and PI had a higher enrichment ratio. Moreover, glycerophosphocholine, PI, PC, and PS had higher enrichment ratios compared with group M and Y. Thus, DHA may improve cognitive abilities by promoting the synthesis process of PLs like PS and PI.

### 3.12. DHA Supplements Improved mRNA Expressions of Lipid Transportation and Metabolism in PFC Tissue

**Figure 5:**
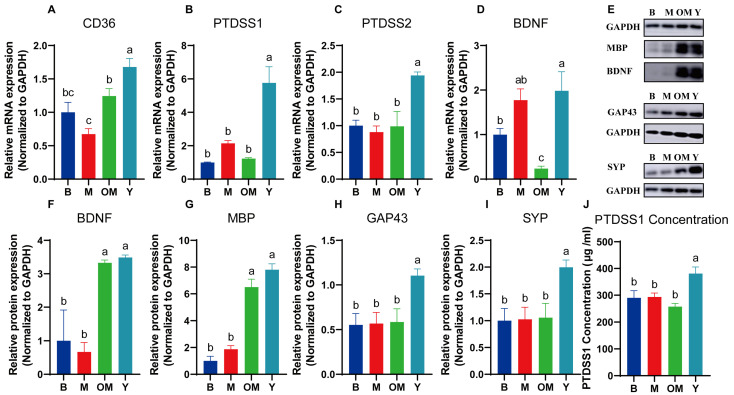
The mRNA expression (**A**–**D**) in PFC tissue from different groups. The protein expression (**E**–**J**) in PFC tissue from different groups. The PTDSS1 concentration (**I**) in PFC tissue from different groups. Data in (**A**–**D**,**J**) are shown as the mean ± SEM, *n* = 6. Data in (**F**–**J**) are shown as the mean ± SEM, *n* = 3. The significance of the differences was estimated by one-way ANOVA and Waller–Duncan. Different letters represent significant differences in protein contents in different lactation periods (*p* < 0.05). GAPDH: Glyceraldehyde-3-phosphate dehydrogenase; BDNF: Brain derived neurotrophic factor; CD36: Thrombospondin receptor; PTDSS1: Phosphatidylserine synthase 1; PTDSS2: Phosphatidylserine synthase 2; BDNF: Brain-Derived Neurotrophic Factor; MBP: Myelin Basic Protein; GAP43: Growth-Associated Protein 43; SYP: Synaptophysin.

Metabolic changes of the cortex are partly driven by a lipid transporter. According to previous studies, DHA is transported with other lipid transporters. Therefore, we examined the mRNA expressions of CD36 (Figure 5A). As expected, the mRNA expressions in group Y were upregulated compared with other groups, and significant differences were found between group Y and OM, revealing that the FO DHA entered into the cortex with higher efficiency compared with milk DHA. Furthermore, we examined the levels of Phosphatidylserine Synthase 1 (PTDSS1) and Phosphatidylserine Synthase 2 (PTDSS2) as a PS synthase whose levels showed that interfering with FO DHA might increase the levels of PS synthesis (Figure 5B,C). To show the consumption, we also examined the content level of PTDSS1 (Figure 5J) in PFC tissue, which exhibited the same trend of mRNA results.

### 3.13. DHA Supplements Improved Synaptic Plasticity in PFC Tissue

As shown in Figure 5E–I, we measured the relative protein expressions of proteins related to brain nutrition and synaptic plasticity. The cortex proteins in group Y showed an obvious increase in protein-relative levels of Brain Derived Neurotrophic Factor (BDNF) (*p* < 0.05) compared with group B. In contrast, these protein levels in group Y also showed a slight increase compared with group Y, revealing that DHA supplements might have better effects for cortex nutrition with FO DHA.

To find more effects on myelin sheath and synaptic plasticity, we also analyzed the protein levels of myelin basic protein (MBP), synaptophysin (SYP), and membrane protein (GAP43) (Figure 5G–I). In most degenerative brain diseases, abnormal levels of MBP mean that a steady state of myelin sheath has been broken. PL is also the main content of cerebroside, which consists of myelin sheath. According to the results of MBP expressions, significant increase levels could be found in group Y in PFC tissue, which reveal that the interference of DHA might increase the development of myelin sheath. The protein expression level of GAP43 and SYP also showed that DHA from FO could significantly promote synaptic plasticity.

## 4. Discussion

PUFAs are indispensable nutrients for the cortex during early childhood, especially for DHA and EPA [42]. Not only are they a critical participant in myelin sheath synthesis, which regulates signal conduction [43], but they can also promote the development of cortex tissue, which can also benefit cognition in children [44,45,46]. It was found that DHA from various origins caused different changes in lipid composition and regulated fat metabolism mode [47,48]. A recent study found that the increase in serum DHA levels of infants whose lactating mothers had a higher consumption of “blue-back fish” and/or “white fish” revealed that FO might be applied for human metabolic mode [49]. Moreover, DHA can also be received from other origin ingredients, like milk, walnuts, and eggs [50,51], which can also potentially promote synaptic plasticity. Such DHA intervention strategies can cause different metabolic levels of related components in the cortex. Through basic recognition, this study used targeted lipidomics to determine the differences in metabolism components for different interference methods. We found that different interfering methods produced related characteristic lipid content, and that group Y had the highest lipid categories with features, as described in the Venn figure (Figure 1I). The correlating heatmap (Figure 1G) analysis of PFC tissue showed that samples in group Y were a unique subcluster, revealing that the DHA from deep-sea FO sharply changed the lipid composition, especially for PLs. Moreover, the chord diagram (Figure 1H) illustrated the great number of categories of characteristic lipids of group Y, which also showed that DHA from deep-sea FO could significantly promote the levels of PC, PE, PI, PS, and ShexCer. Thus, this study used targeted lipidomics to evaluate PS levels with DHA interference from various origins for the first time. Moreover, we also performed a pathway analysis and network analysis to find the related pathway in which DHA could regulate cortex unsaturated fatty acid synthesis. The results show that DHA supplements effectively upregulated the metabolism of lipids and signal conduction in the cortex, which also influences synaptic functions. In the enrichment analysis, we found that PS and PI had different enrichment ratios, which revealed that they might have fluctuating content levels with different DHA interfering strategies. Furthermore, some researchers have found that PS dysregulation may cause axonal stress that can threaten the functions of signal conduction [52]. Furthermore, DHA-PS could effectively improve cell morphology and promote the restoration of neural network structure [53].

Previous studies have found the differences of major PLs metabolites with DHA interference in brain tissue, particularly in Ether, PC, PS, and ShexCer. Among the vital contents, PC permits the selective click labeling of an organelle [54]. The results of the lipidomics analysis show that the content levels of SM, PS, and PC in group Y were significantly higher than group B and group M. That is, the results of network analysis filtered PS and PC as the strongest interacting metabolite classifications. For these specific reasons, children’s lipid metabolic modes may highly match with FO DHA, especially for cortex tissue. On the basis of promoting cell morphology in the brain, we hypothesized that synaptic plasticity exerts influence by increasing the level of PS with DHA interference. Moreover, previous studies have shown that PUFAs like DHA and EPA interference may promote the membrane functions for neuron membranes, which improves the synaptic correlation and synthesis process of myelin sheath. The myelin sheath’s main function is to protect the signal conduction between neurons. DHA may improve cognitive abilities, information integration, and sleep regulation through such a mechanism. We found that group Y had significantly higher content levels of PC, PI, PS, and SM than group M and group B. As the main component of myelin sheath, higher SM levels promote the accurate ratio of signal conduction, which also ensures the normal regulation activities in the cortex. Furthermore, a study found that DHA-PS could effectively alleviate brain degenerative disease [55]. Therefore, we chose PS as our research focus and examined the mRNA expressions of CD36, PTDSS1, and PTDSS2, as well as tissue concentration of PTDSS1, which is the main PS synthase [56]. The results show that FO DHA might have a higher transporting ratio into the brain and transfer ratio from DHA to PS to promote synaptic plasticity in PFC.

We also observed an increase in the content levels of PLs in the PFC tissue from group Y, whose PS levels were higher than other groups. PS is one of the important components of cell and organelle membranes, and it is the lipid mediator for organelle fusion with the plasma membrane [57]. Then, we showed that the absorption of DHA from FO into infantile pigs induced the promotion of myelin basic protein, and synaptic plasticity-related proteins in the PFC tissues of BPs were the focus in this context. We intend on doing further research on the interference influences on the development of synapse and myelin sheath, which are indicators to estimate cognitive function.

In sum, we found that DHA supplements with FO could significantly upregulate PS synthetase, meaning that a DHA supplement could effectively promote brain functions by raising PS levels. According to previous studies, PS has specific features in the cortex [58], which may promote the cognitive functions of the elderly [59]. These results show that DHA interference enhanced DHA intake levels and synaptic plasticity in PFC tissue. Without a doubt, such DHA interference could promote synapse development in PFC tissue and accelerate the formation of myelin sheath and synapse by promoting the synthesis of PS. Supplemented DHA is transported through the BBB, and it actually improves the synthesis of PS, which promotes the functions of receptors and related proteins in the membrane and myelin sheath, whose main function is regulating signal conduction. These data may raise more questions, mainly about the relationship between synaptic plasticity and DHA metabolism modes.

It is difficult to assess the real effective metabolites of DHA and PS in the brain. Given the whole levels of species in PFC tissue, we can hypothesize that the synapse cell membrane and myelin-sheath synthesis pathways are significantly upregulated with DHA interference from fish origins. Because of the alternation of PS and other PLs, we can suitably assume that the increase in related indicators is due to different combined interventions of DHA. Even though we found the biomarkers for DHA metabolism in PFC tissue, we still aimed to seek the triggering metabolites for the synaptic plasticity in the PFC. With DHA intake from various origins, it was interesting to find that different interference strategies made different lipid compositions, which determined the development situation for myelin sheath and synapse. The effects of the DHA and milk combination increased the SM level, which also upregulates the expression of SYP, which is the marker for synaptic plasticity. Hence, we assumed that DHA metabolism in the cortex may include the specific precursors that promote the synaptic plasticity with the supplement of DHA from FO.

Related molecular mechanisms of DHA metabolism and synaptic plasticity remain elusive and complex. In this paper, we observed a positive correlation between DHA supplements and synapse. Indeed, irrational interference strategies may not meet our expectations. However, we focused on PS, which can be synthesized from DHA, which improves synapse functions in the cortex. It would be interesting to analyze the association between interference modes and myelin-sheath synthesis in microglia and astrocytes.

We acknowledge that the evidence of this study lacked morphological indicators. Moreover, the results of lipidomics, RT-qPCR, and western blot are sufficient to conclude that DHA interference from FO can effectively promote the synthesis of PS and synaptic plasticity. However, we acknowledge that the lack of synapse structure data was a limitation of this study. However, the relative expressions of SYP and GAP43 suggest that DHA from FO may promote synaptic plasticity in the pre-frontal cortex tissue. The strength of this study was that the targeted lipidomics technology represented the first usage of a pig model to analyze the effect of DHA intervention from various sources. Also, we examined the lipid content levels of PFC tissue to analyze the lipid-content changes of different interfering methods.

## 5. Conclusions

In conclusion, in the investigated PFC tissue, a total of more than 80 related negative ion lipids were identified and quantified. The results of this study reveal new insights into the physiological lipid metabolisms, especially for those PLs related to the PFC and synaptic plasticity. Then, this study first analyzed the main metabolites of DHA intervention from various origins in the cortex. It was revealed that DHA played a specific role in the synaptic plasticity and synthesis of the myelin sheath in the early life of BPs, which have similar features to human beings, by changing the lipid composition in the cortex, compared with other lipids in soybean oil and blended oil. We found that DHA supplements from FO could effectively upregulate the synthesis of PS to promote synaptic plasticity in the PFC. Our findings provide further insight into the intervention strategies of DHA, a new evaluation method of DHA intervention influences on PFC, and design for related functional food in particular.

## Figures and Tables

**Table 1:** Related specific components of DHA-supplemented milk.

Name	Calorie (kcal/100 mL)	Protein (g/100 mL)	Carbohydrate (g/100 mL)	Fat (g/100 mL)	DHA (mg/100 mL)
Milk without DHA (Group B)	73.8	3.6	5.0	4.4	0
Model milk (Group M)	73.8	3.6	5.0	4.4	0
Naturally supplemented milk (Group OM)	71.2	3.6	4.8	4.2	20
FO-supplemented milk (Group Y)	73.8	3.6	5.0	4.4	20

The intaking DHA standard of animals in group OM and Y was 11 mg/kg/d. The intake volume of liquid milk was 55 g/kg/day, while the intake volume of oil was 7.5 g/kg/day. All the animals were mixed-fed with milk and DHA-free milk.

**Table 2:** Feed formulation.

Name	Core (%)	Soybean Meal (%)	Premix (%)	DHA	Calorie (kcal/kg)
Feed	76	20	4	0	3270

**Table 3:** Related program of linear gradient elution.

Time (min)	%A	%B
0	60	40
1.5	15	85
10.5	15	85
14	15	85
14.1	0	100
15.1	0	100
15.3	60	40

Mobile phase A: 10 mM ammonium formate: water/acetonitrile (6:4). Mobile phase B: 10 mM ammonium formate: isopropanol/acetonitrile (9:1).

**Table 4:** Setting parameters of electric spray ionization interface.

Setting Items	Setting Value
Ion Source	Point Jet Ionization Interface
Drying Machine Temperature	300 °C
Nebulizer Pressure	35 psig
Sheath Temperature	35 °C
Sheath Flow Rate	12 L/min
Crusher Voltage	140 V
Capillary Voltage	3500 V
Collection Rate	3 Spectrum/s
Scanning Rate	100–1700 *m*/*z*

**Table 5:** Primer Sequences of Each Gene in this Study.

Name	Oligo	Primer Sequence
GAPDH	F	TCGGAGTGAACGGATTTGGC
	R	TGACAAGCTTCCCGTTCTCC
PTDSS1	F	TCAGCCTCATGTACTTCGCC
	R	AGTTCACAGCATACTCCATTGGG
PTDSS2	F	GAACACCTTCTTCTGGCGGG
	R	CTCTTGGTGTTGTAGGCCGT
BDNF	F	GAGTCTGGGGATTTCGGGG
	R	CTCACCTGGTGGAACTTTTCAG
CD36	F	TGACCCAGCACTTGAAGCAA
	R	TCCAAGAGGTATGCTTCTTTTCCA

GAPDH: glyceraldehyde-3-phosphate dehydrogenase; PTDSS1: phosphatidylserine synthase 1; PTDSS2: phosphatidylserine synthase 2; BDNF: brain derived neurotrophic factor; CD36: thrombospondin receptor.

**Table 6:** Body and brain weight for each group.

Name	Body Weight (kg)	Brain Weight (g)
Group B	15.70 ± 1.06	63.66 ± 3.49
Group M	14.12 ± 1.51	56.09 ± 8.87
Group OM	15.14 ± 0.97	60.09 ± 2.45
Group Y	14.15 ± 3.11	58.13 ± 6.59

**Table 7:** Network analysis of lipid metabolites in PFC tissue. B vs. Y; M vs. Y; OM vs. Y. Each node represents a lipid metabolite, and each edge represents the interaction between metabolites.

B vs. Y		M vs. Y		OM vs. Y	
Node	Degree	Node	Degree	Node	Degree
PI 18:0_20:3	9	PE 18:0_22:4	7	SHexCer d36:1	9
SM d36:1	7	PS 18:0_20:3	7	SHexCer d38:2	8
PS 20:1_22:6	7	PC 16:0_18:1	6	PC 18:0_18:1	7
PE 18:0_22:4	6	SHexCer d38:2	6	PE 18:1_22:6	6
PS 18:0_20:3	6	PI 18:0_20:4	6	PS 18:0_20:3	6

## Data Availability

Data presented in this study are available on request from the corresponding author.

## Abbreviations

BBB: blood-brain barrier; DHA, docosahexaenoic acid; EPA, eicosapentaenoic acid; ether: ether-related lipids; LC-MS: liquid chromatography and mass spectrometry technique, LPC: lysophosphatidylcholine; LPE: lysophosphatidylethanolamine; PUFA: polyunsaturated fatty acid; PC: phosphatidylcholine; PE: phosphatidylethanolamine; PFC: prefrontal-cortex; PI: phosphatidylinositol; PS: phosphatidylserine; SHexCer: Sulfated hexosylceramide; SM: sphingomyelin; BDNF: brain-derived neurotrophic factor; CD36: thrombospondin receptor; GAPDH: glyceraldehyde-3-phosphate dehydrogenase; MBP: myelin basic protein; PTDSS1: phosphatidylserine synthase 1; PTDSS2: phosphatidylserine synthase 2; SYP: synaptophysin.
